# Monocyte Subsets Have Distinct Patterns of Tetraspanin Expression and Different Capacities to Form Multinucleate Giant Cells

**DOI:** 10.3389/fimmu.2018.01247

**Published:** 2018-06-08

**Authors:** Thomas C. Champion, Lynda J. Partridge, Siew-Min Ong, Benoit Malleret, Siew-Cheng Wong, Peter N. Monk

**Affiliations:** ^1^Department of Infection, Immunity and Cardiovascular Disease, University of Sheffield, Sheffield, United Kingdom; ^2^Singapore Immunology Network, Agency for Science, Technology and Research, Singapore, Singapore; ^3^Department of Molecular Biology and Biotechnology, University of Sheffield, Sheffield, United Kingdom; ^4^Department of Microbiology and Immunology, Yong Loo Lin School of Medicine, National University of Singapore, National University Health System, Singapore, Singapore

**Keywords:** monocyte, tetraspanin, cd9, fusion, monocyte subsets

## Abstract

Monocytes are able to undergo homotypic fusion to produce different types of multinucleated giant cells, such as Langhans giant cells in response to *M. tuberculosis* infection or foreign body giant cells in response to implanted biomaterials. Monocyte fusion is highly coordinated and complex, with various soluble, intracellular, and cell-surface components mediating different stages of the process. Tetraspanins, such as CD9, CD63, and CD81, are known to be involved in cell:cell fusion and have been suggested to play a role in regulating homotypic monocyte fusion. However, peripheral human monocytes are not homogenous: they exist as a heterogeneous population consisting of three subsets, classical (CD14^++^CD16^−^), intermediate (CD14^++^CD16^+^), and non-classical (CD14^+^CD16^+^), at steady state. During infection with mycobacteria, the circulating populations of intermediate and non-classical monocytes increase, suggesting they may play a role in the disease outcome. Human monocytes were separated into subsets and then induced to fuse using concanavalin A. The intermediate monocytes were able to fuse faster and form significantly larger giant cells than the other subsets. When antibodies targeting tetraspanins were added, the intermediate monocytes responded to anti-CD63 by forming smaller giant cells, suggesting an involvement of tetraspanins in fusion for at least this subset. However, the expression of fusion-associated tetraspanins on monocyte subsets did not correlate with the extent of fusion or with the inhibition by tetraspanin antibody. We also identified a CD9^High^ and a CD9^Low^ monocyte population within the classical subset. The CD9^High^ classical monocytes expressed higher levels of tetraspanin CD151 compared to CD9^Low^ classical monocytes but the CD9^High^ classical subset did not exhibit greater potential to fuse and the role of these cells in immunity remains unknown. With the exception of dendrocyte-expressed seven transmembrane protein, which was expressed at higher levels on the intermediate monocyte subset, the expression of fusion-related proteins between the subsets did not clearly correlate with their ability to fuse. We also did not observe any clear correlation between giant cell formation and the expression of pro-inflammatory or fusogenic cytokines. Although tetraspanin expression appears to be important for the fusion of intermediate monocytes, the control of multinucleate giant cell formation remains obscure.

## Introduction

Human monocytes are able to migrate from the bloodstream into the tissues and differentiate into macrophages and monocyte-derived dendritic cells (1). They are important in defense against various pathogens (2) but are also implicated in autoimmune and inflammatory diseases (3). Blood monocytes are heterogeneous and three subsets have been defined: classical (Cl, CD14^++^CD16^−^), intermediate (Int, CD14^++^CD16^+^), and non-classical (NCl, CD14^+^CD16^+^), comprising ~85, 5, and 10% of the total, respectively (3, 4). Investigation of the maturation and differentiation kinetics of labeled human monocytes *in vivo* suggests that they mature from Cl to Int and then to NCl (5, 6). The subsets differ in their gene expression profiles, cell surface markers, and cytokine secretion (7–11). The blood populations of the Int and NCl have been observed to be increased in patients with tuberculosis (12) and rheumatoid arthritis (13), whereas Int numbers are increased in various other inflammatory conditions, including Crohn’s disease (14), sarcoidosis (15), and cardiac disease (16, 17).

Under certain circumstances, monocytes and macrophages are able to fuse to form multinucleated giant cells (MGC), such as the osteoclast MGC that remodel and maintain bone homeostasis (18). Monocytes can form inflammatory MGC, such as Langhans giant cells (LGC), in response to *M. tuberculosis* infections during granuloma formation around infected macrophages (19). Monocytes can also fuse in response to non-phagocytosable foreign material such as medical implants, forming foreign body giant cells (FBGC) (20).

The mechanism of monocyte fusion is still largely unknown and only a handful of essential proteins have been identified (21, 22). Furthermore, LGC and FBGC formation appears to be initiated by different cytokines, IFNγ and IL-4, respectively, which could suggest that they coordinate fusion through multiple signal transduction pathways (23, 24). Monocytes activated by fusogenic stimuli secrete chemokines, such as CCL2 and CCL3, upregulate cell–cell adhesion proteins (LFA-1, ICAM-1, and E-cadherin) (25) and fusion-facilitating proteins, such as CD200 (26), SIRPα/CD172a/MFR (27), CD47 (28), CD36 (29), CD62E (E-selectin) (30), matrix metallopeptidase 9 (MMP9) (31), and dendrocyte-expressed seven transmembrane protein (DC-STAMP) (32, 33).

The tetraspanin family of membrane proteins has been implicated in the regulation of several different types of cell–cell fusion, including CD9, CD81, and CD151 in sperm–egg interactions (34), CD9 and CD81 in muscle cell fusion (35), CD82 in HTLV-1 syncytial formation (36) and CD9 in HIV-1-induced cell fusion (37). Osteoclast formation is known to be regulated by CD9, Tspan-5, and Tspan13 (38, 39). In experimental systems using concanavalin A (ConA)-induced fusion, anti-tetraspanin antibodies against CD9, CD81, CD151, and CD63 have been shown to inhibit or enhance the formation of MGC (40–42). Importantly, many of the fusion regulatory proteins implicated in MGC formation have been shown to be associated with tetraspanins in the plasma membrane (43).

Recently, CD9, CD53, CD63, and CD81 were shown to be expressed differently on the three monocyte subsets (44), indicating that subsets may have different fusion behaviors. In this study, we have investigated the propensities of the monocyte subsets for fusion, and attempted to correlate this with the expression of a group of fusion-related tetraspanins, fusion proteins, and cytokines. Further understanding of the contribution of monocyte subsets to fusion and the role tetraspanins play in the fusion process may help develop treatments for granulomatous diseases such as tuberculosis and inhibit foreign body reactions during medical implant rejection.

## Materials and Methods

### Cells

All experiments used human blood monocytes collected in EDTA. For experiments using purified monocyte subsets, cells were obtained from apheresis cones donated by anonymous platelet donors in Singapore. Blood samples and experimental procedures were approved by the Institutional Review Board, Singapore, in accordance to guidelines of the Health Science Authority of Singapore (Reference code: NUS-IRB10-250). Informed written consent was obtained from participants for this study in accordance with the Declaration of Helsinki. Apheresis cones contain approximately 400–1,200 × 10^6^ cells per cone, of which ~68% are lymphocytes, ~25% monocytes, ~5% neutrophils, ~2% basophils, and <1% eosinophils (45).

### Monocyte Purification

Human blood from apheresis cones was diluted 1:1 in Dulbecco’s phosphate buffered saline without Ca^2+^ and Mg^2+^ (Lonza). Diluted blood was separated on Ficoll-Paque PLUS (GE Healthcare Life Sciences) by centrifugation. The PBMC layer was removed and washed with saline to remove platelets. Red blood cells were lysed and cell number and viability determined by counting in the presence of Trypan Blue (Sigma-Aldrich). Total monocytes were positively selected using anti-CD14-beads according to manufacturer’s instructions (Miltenyi Biotec). The purity as determined by flow cytometry, and viability by Trypan blue exclusion were consistently >90%. In some cases, monocytes were also purified by adherence to plastic, as described previously (41). Monocytes for subsequent subset fractionation were first enriched by depleting non-monocytic cells using magnet-activated cell sorting (MACS) with anti-CD3 and anti-CD19-beads, according to manufacturer’s instructions (Miltenyi Biotec). MACS-enriched monocytes contained typically 69% monocytes. For FACS purification of monocyte subsets, a cocktail containing anti-CD14-efluor450 (eBioscience), anti-CD16-FITC (Miltenyi Biotec), and anti-CD56-APC (BD Biosciences) was added to the MACS-enriched total monocytes. Contaminating NK cells were excluded and monocyte subsets: Cl (~80%; CD14^++^CD16^−^), Int (~8%; CD14^++^CD16^+^), and NCl (~11%; CD14^+^CD16^++^) were gated based on the gating strategy shown in Figure S1 in Supplementary Material. To maintain reproducibility, subsets were always gated with equal sized square gates with perpendicular borders. A post-sort check was conducted in every subset to ensure that the purity of each subset was ≥90%.

### Fusion Assays

FACS-purified monocyte subsets were seeded at 1.5 × 10^5^ cells per 31.65 mm^2^ well to give a cell density of 4,739 monocytes mm^−2^. Within an hour of seeding, ConA from *Canavalia ensiformis* (Sigma-Aldrich) was added at 10 µg ml^−1^ in IMDM (Lonza) containing human AB Serum (Innovative Research, Inc., IPLA-SERAB) and penicillin/streptomycin (Biological Industries). Monocyte subsets were incubated for up to 72 h at 37°C in 5% CO_2_ for all fusion assays. The supernatant from each well was collected and stored at −80°C for cytokine measurements. Cells were stained with nucleus/actin staining solution containing 3 µg ml^−1^ DAPI (ThermoFisher) and 1 µg ml^−1^ Phalloidin-TRITC (ThermoFisher) overnight at 4°C in the dark. The cells were then fixed and imaged with an Olympus IX83 inverted microscope running MetaMorph for Olympus imaging software (Olympus, UK). MGC were identified from the image Stack on FIJI ImageJ and freehand outlines were drawn around each MGC (defined as cells with ≥3 nuclei) to make Region Of Interest coordinates that could be saved alongside the Stack files. The DAPI stack and ROI list file were loaded in ImageJ before using a selection on user-generated macros to count the nuclei per MGC, MGC area and the total number of nuclei per field. MGC types were designated using the criteria outlined in Figure S2 in Supplementary Material. LGC and FBGC are known types of MGC but a third category was also detected in our studies, which we termed the syncytial giant cell (SGC). SGC are characterized by having no clear organization of nuclei and with patchy staining for polymerized actin (Figure S2 in Supplementary Material). In all cases, the total nuclei counted in single and fused cells were much lower than the number originally plated. The missing nuclei were designated as “Detached Cells” but the fate of these cells was not investigated further.

### Measurement of Tetraspanin and Fusion Protein Expression by Flow Cytometry

A 10-marker panel was developed that could identify the three monocyte subsets, quantify the expression of seven tetraspanins and detect cell viability all in one sample (Table S1A in Supplementary Material). The panel consisted of a LIVE/DEAD Blue dye, two monocyte subset markers (anti-CD14- PE-CF594 and anti-CD16- PE-Vio770) and tetraspanins (anti-CD9-Biotin, anti-CD37-APC, anti-CD53-CF405M, anti-CD63-PerCP, anti-CD81-Alexa Fluor 700, anti-CD82-PE, anti-CD151-FITC). Strepavidin-APC-Cy7 was used as the secondary reporter for CD9-Biotin. To detect changes after ConA treatment, adherent monocytes were treated with or without ConA for 4 h (at which point monocyte fusion can be observed), and then harvested by scraping prior to antibody staining, as above. A compensation matrix was generated on FACSDiva software using negative control or capture anti-mouse Fc compensation beads for all fluorophore combinations. In separate experiments, fusion protein antibodies were used individually on freshly isolated monocytes, with a FITC-labeled secondary antibody, using the CD14/CD16 antibody pair to distinguish subsets. In all cases, antibodies were individually titrated to ascertain the concentration for optimum binding and compared to an appropriate isotype control antibody. Flow acquisition was performed on a BD LSR II.

### Median Nuclei per MGC

The number of nuclei per MGC had a positive skew whereby smaller (3–8 nuclei) MGC were far more common than larger (≥20 nuclei) MGC and so the median was used to describe the average size of a giant cell in any given condition.

### Fusion Index (FI)

Fusion index expresses the fusion of cells as the ratio of nuclei inside fused cells with ≥3 nuclei to the total number of nuclei counted and expressed as a percentage.

### Cytokine Assays

The supernatants collected at 24, 48, and 72 h from the fusion studies were stored at −80°C before analysis for CCL2 (MCP-1), CCL3 (MIP-1α), RANTES, IL-1α, IL-1β, TNFα, IL-6, IL-17A, IL-4, IL-10, IL-13, GM-CSF, IL-3, IFNγ, and VEGF, using Luminex^®^ xMAP^®^ technology and customized human 9- and 15-plex kits (Merck Millipore) with DropArray™-bead plates (Curiox).

### Scanning Electron Microscopy (SEM)

For imaging by SEM, sorted cells were allowed to adhere for 15 min at room temperature to glass coverslips pretreated with poly-l-lysine (Sigma), then were fixed for 1 h at room temperature in 2.5% (w/v) glutaraldehyde and 0.1 M phosphate buffer (pH 7.4) and were washed twice in PBS. After fixation for 1 h at room temperature with 1% (w/v) osmium tetroxide (Ted Pella), cells were washed in deionized water and dehydrated with a graded series of ethanol immersions from 25 to 100%, and were dried to the critical point (CPD 030; Bal-Tec). The glass coverslip was then laid on adhesive film on a scanning electron microscope sample holder and was firmly touched with an adhesive sample holder. The surface on which the cells were deposited, as well as the adhesive surface, were both coated with 5 nm of gold in a high-vacuum sputtering device (SCD005 sputter coater; Bal-Tec). The coated samples were examined with a field emission scanning electron microscope (JSM-6701F; JEOL) at an acceleration voltage of 8 kV with the in-lens secondary electron detector. Fluorescence and brightfield images were also taken and collaged into larger map images using ImageJ FIJI. The brightfield map was compared with the low magnification SEM images to identify the location of the high magnification SEM images. The appropriate high magnification SEM images and 20× magnification fluorescent images were then matched, cropped, and merged using ImageJ.

### Statistical Analyses

All statistical analyses were performed with GraphPad Prism v6.04 and the appropriate tests are noted in the legend of each figure. In all figures the data value represents the number (*n*) of different donor repeats in the experiment, and the SEM is reported where *n* ≥ 3, except where stated. All fluorescence-based values [flow cytometry median fluorescence intensity (MFI)] were log-transformed before statistical analysis. *p ≤ 0.05, **p ≤ 0.01, ***p ≤ 0.001, ****p ≤ 0.0001.

## Results

### Monocyte Subsets and MGC Formation

Human blood monocytes were first negatively selected by removing non-monocytic cells using MACS and then subjected to positive selection for individual subsets using stringent gating based on anti-CD14 and anti-CD16 antibody binding (Figure S1 in Supplementary Material).

**Figure 1:**
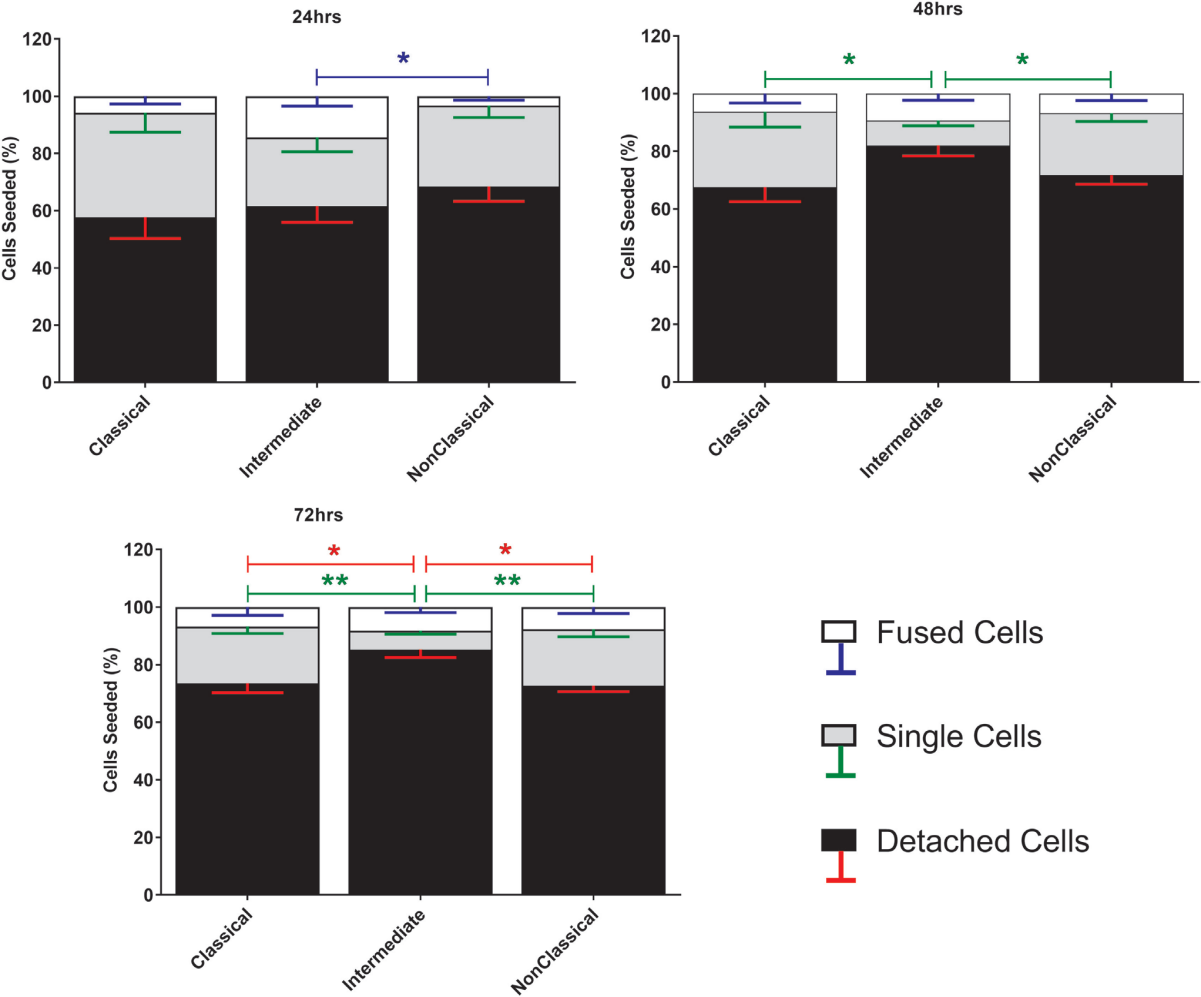
Cell fate during ConA-induced fusion varies between monocyte subsets. The fate of sorted monocyte subsets was determined by counting nuclei at 24, 48, and 72 h and expressed as a percentage of the cell numbers originally plated. Bars represent means ± SEM, *n* = 8. Significance was tested with a Kruskal–Wallis test with a Dunn’s multiple comparisons test comparing the means of the same fate and time point against the other subsets. Black bars/red error bars: detached cells, gray bars/green error bars: single cells and white bars/blue error bars: fused cells with >3 nuclei.

Fusion was induced using ConA, a lectin known to stimulate cell fusion in diverse cell types, e.g., *Drosophila* somatic cells (46). The exact mechanism of ConA facilitated fusion is currently unknown. However, it has been shown that ConA triggers a release of fusion initiating cytokines from mouse macrophages, such as IFNγ, TNF-α, IL-1β, and IL-4 (47). The behavior of the different subsets during ConA stimulation was determined by counting stained nuclei to provide a measure of the proportions of single and fused cells where the latter refers to cells with >3 nuclei (Figure 1). Interestingly, the majority of the monocytes were lost after 72 h, presumably due to detachment and/or cell death. Int monocytes fused more rapidly than Cl and NCl subsets, but were also significantly more likely to be dead/detached by 48 and 72 h. Cl monocytes were significantly less likely to fuse than either of the other two subsets, although the differences between subsets became less pronounced over time.

**Figure 2:**
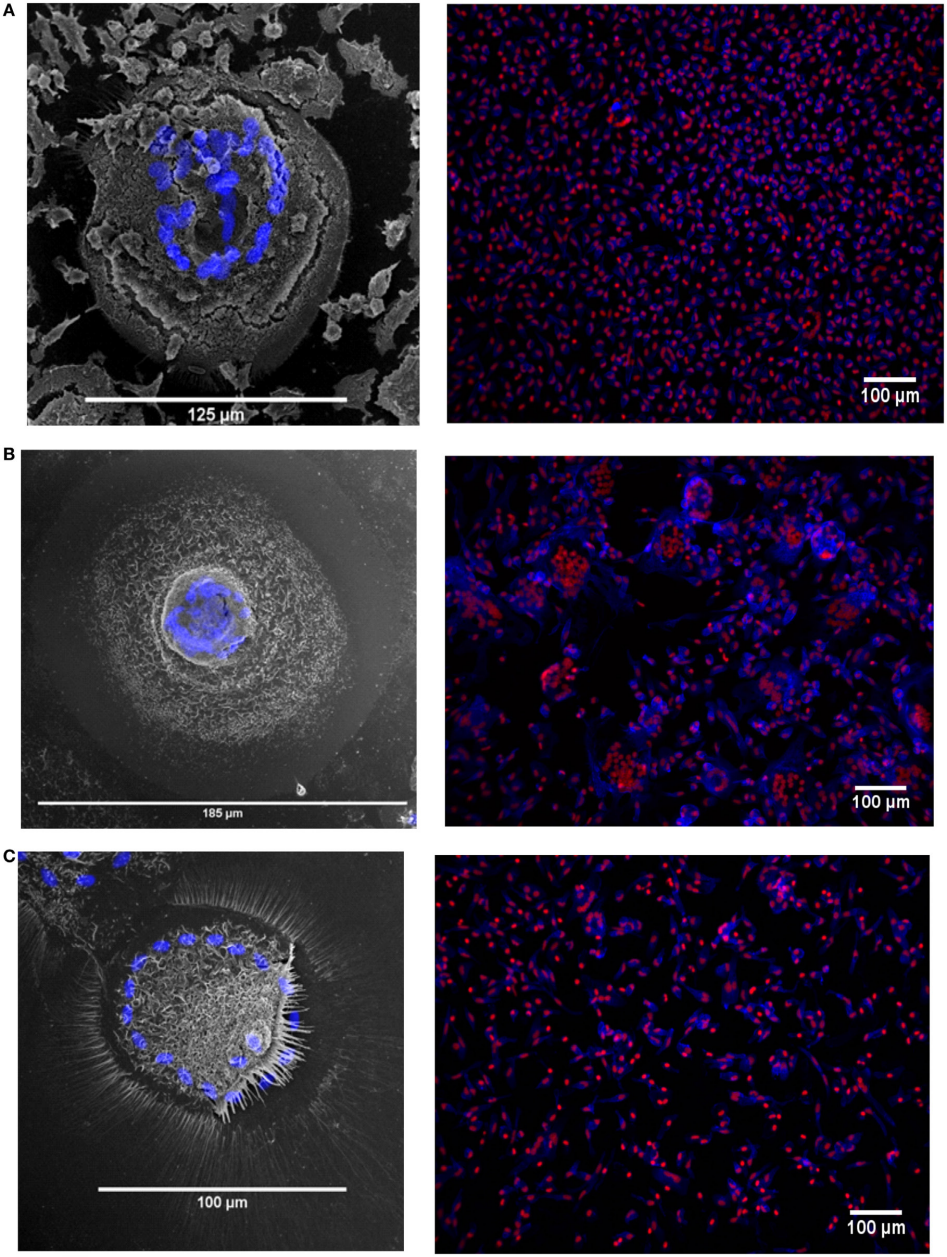
Fusion in different monocyte subsets imaged by tandem fluorescent scanning electron microscopy (SEM) and wide field fluorescence microscopy. **(A–C)** left panels. monocyte-derived giant cell (MGC) were generated by 72-h concanavalin A (ConA) treatment of FACS-sorted monocyte subsets, stained with Hoechst and then raster-scanned so that the MGC imaged in SEM could be located and the nuclear channel overlaid onto the image. Nuclei shown in blue. **(A–C)** right panels. Three representative montages containing images taken of each of the monocyte subsets from one donor after 72 h ConA treatment. Blue = F-actin, Red = nuclei. **(A)** classical, **(B)** intermediate, **(C)** non-classical subset-derived MGC.

**Figure 3:**
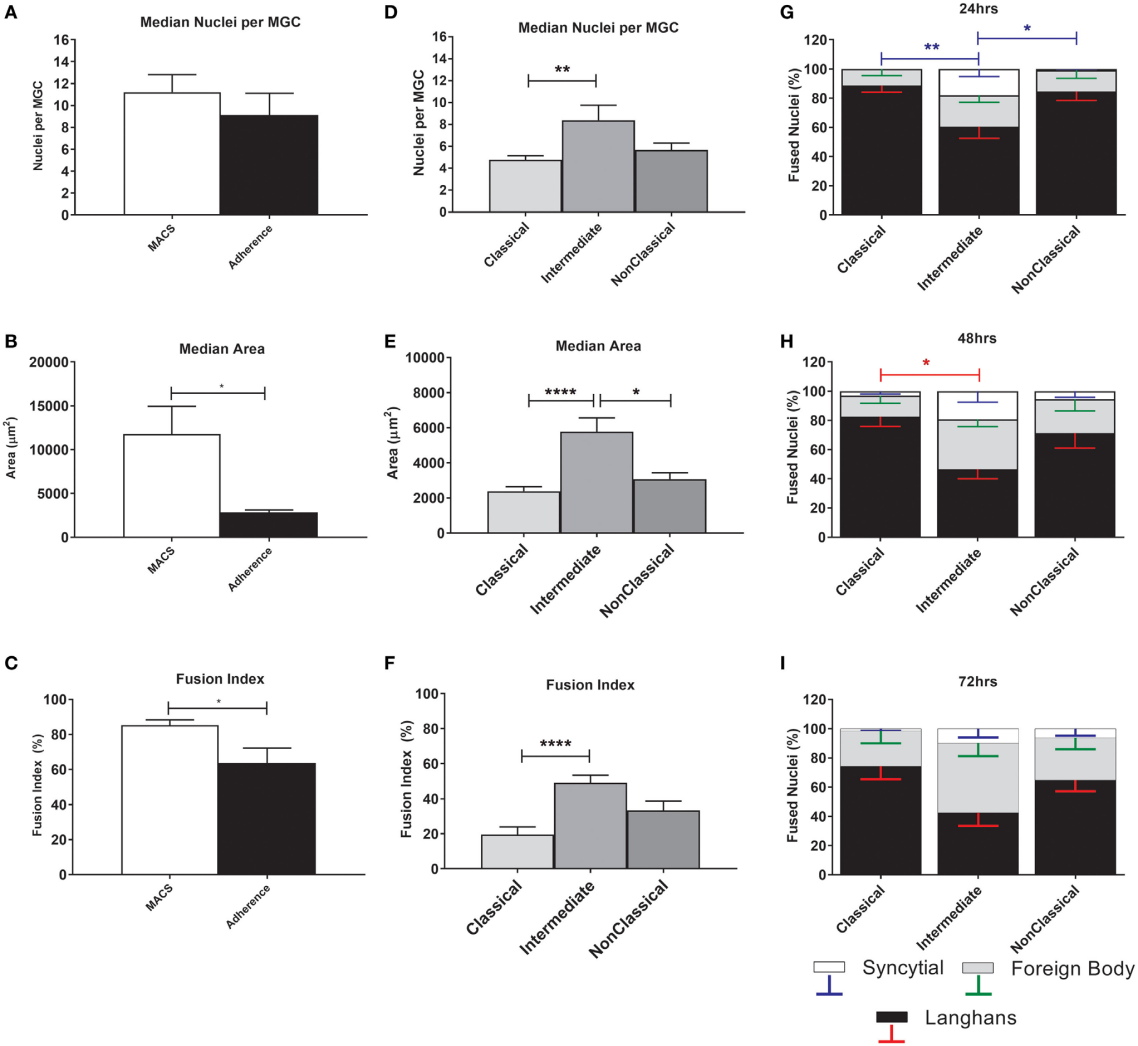
Fusion parameters vary between monocyte purification method and between subsets. **(A–C)** Unfractionated monocytes, purified by magnet-activated cell sorting (MACS) or adherence were incubated for 72 h with concanavalin A (ConA) to induce fusion. After fluorescence imaging, three parameters of monocyte-derived giant cell (MGC) were recorded: median number of nuclei/MGC, fusion index (FI), and median area occupied by each MGC. Significance was tested using an unpaired *t*-test. **(D–F)** Monocytes sorted into subsets by FACS were incubated for 72 h with ConA to induce fusion. After fluorescence imaging, three parameters of MGC were recorded: median number of nuclei/MGC, FI and median area occupied by each MGC. Significance was tested with a Kruskal–Wallis test with a Dunn’s multiple comparisons test. **(G–I)** After 24, 48, and 72 h incubation with ConA, the proportions of each type of MGC were recorded and presented as a percentage of the total fused nuclei counted. Bars represent means ± SEM, *n* = 8. Significance was tested with a Kruskal–Wallis test with a Dunn’ multiple comparisons test comparing the means of the same MGC type within the same time point against the other subset means. Black bars/red error bars: Langhans giant cell, gray bars/green error bars: FBGC and white bars/blue error bars: syncitial giant cell (SGC).

The MGC were classified as LGC, FBGC, or SGC based on the arrangement of nuclei within each MGC according to the criteria shown in Figure S2 in Supplementary Material. Representative wide field and tandem fluorescence-SEM images of fused monocytes formed after 72 h are shown for each subset in Figure 2. There are distinct differences in the sizes and morphologies of the MGC (Figure 2), suggesting subset-specific factors in MGC formation. Interestingly, the monocyte purification method, i.e. adherence or positive purification by MACS using anti-CD14 strongly affected MGC formation in response to ConA. Despite a similar median number of nuclei observed per MGC, MACS-purified monocytes formed significantly larger MGC than adherence-purified monocytes, as determined by the median area and FI (Figures 3A–C).

The physical parameters of the MGC formed by monocyte subsets were assessed at 72 h. FI is significantly higher for Int relative to Cl monocytes, as is the median number of nuclei per MGC (Figures 3D–F), while Int and NCl subsets were similar for both measurements. Interestingly, the median area covered by each MGC is higher for Int monocytes relative to the other two subsets, perhaps related to the higher percentages of FBGC and SGC observed in Int cultures (Figures 3G,H). At 24 h, the Int monocytes formed significantly more SGC (Figure 3G) whereas at 48 h of ConA stimulation, they were significantly less likely to form LGC than Cl monocytes (Figure 3H). By 72 h, no significant difference in the types of MGC formed was observed between the subsets (Figure 3I). Thus, there are quantitative differences between the MGC formed by the subsets, in terms of the kinetics of fusion, the sizes, and morphologies of the MGC formed.

### Tetraspanin Expression on Monocyte Subsets

**Figure 4:**
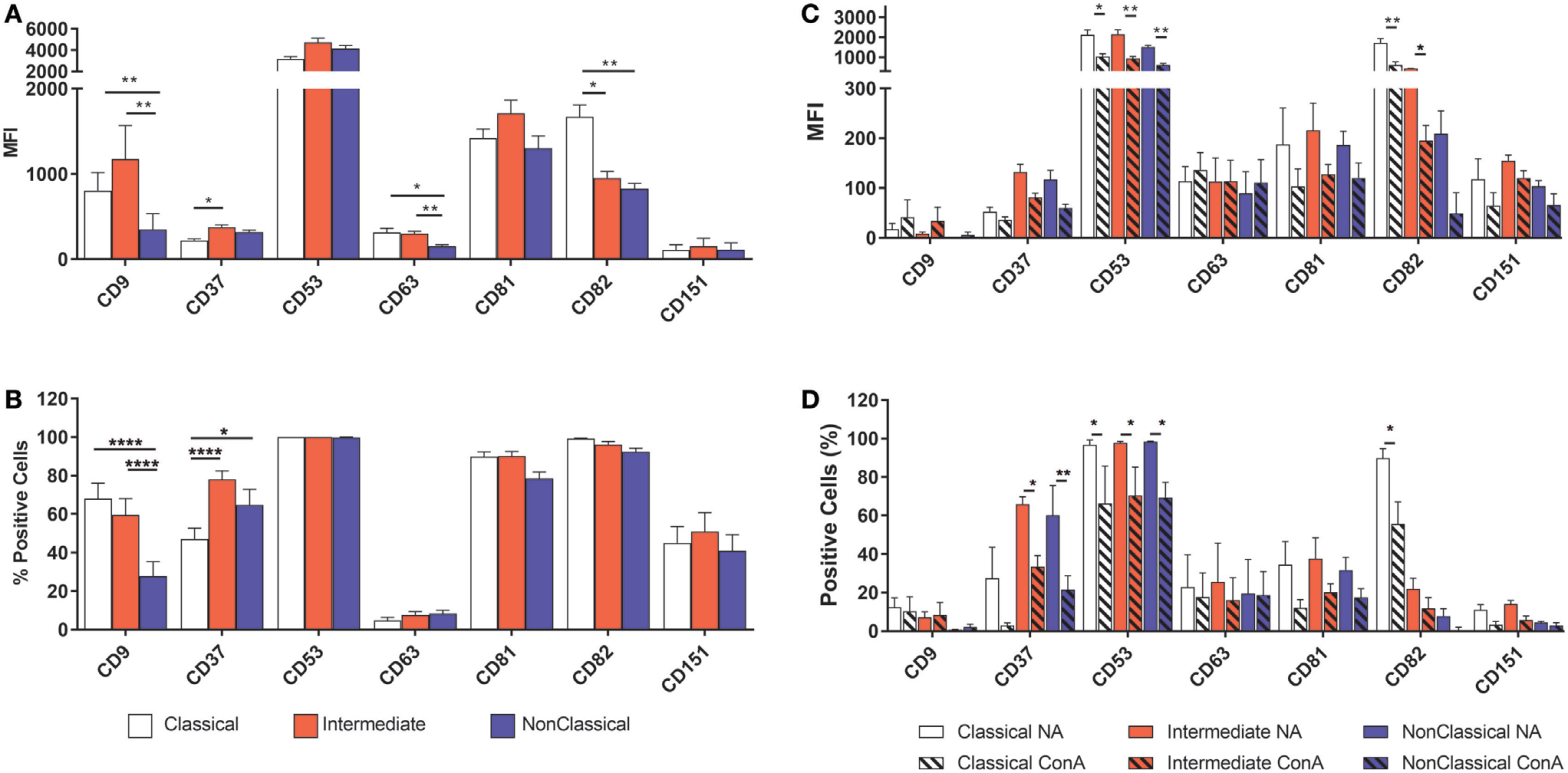
Tetraspanin expression varies between monocyte subsets. Monocytes were either freshly sorted into subsets by FACS, or were purified then allowed to adhere, incubated for 4 h with concanavalin A (ConA) to induce fusion and then harvested, before being tested for the expression of a panel of common myeloid cell tetraspanins using flow cytometry. **(A)** Freshly purified monocyte subsets, expression level per cell (MFI). **(B)** Freshly purified monocyte subsets, percentage of the cell population with expression above isotype control binding levels. **(C)** Adherent, ConA treated monocytes, expression level per cell (MFI). **(D)** Adherent, ConA treated monocytes, percentage of the cell population with expression above isotype control binding levels. The data are the means ± SEM of monocytes from four donors. For **(A,B)**, significance was tested by two-way ANOVA and a Tukey multiple comparison test. For **(C,D)**, significance was tested with multiple *t* tests with Benjami, Krieger, and Yekutieli false discovery rate approach and Holme–Sidak multiple comparisons.

**Figure 5:**
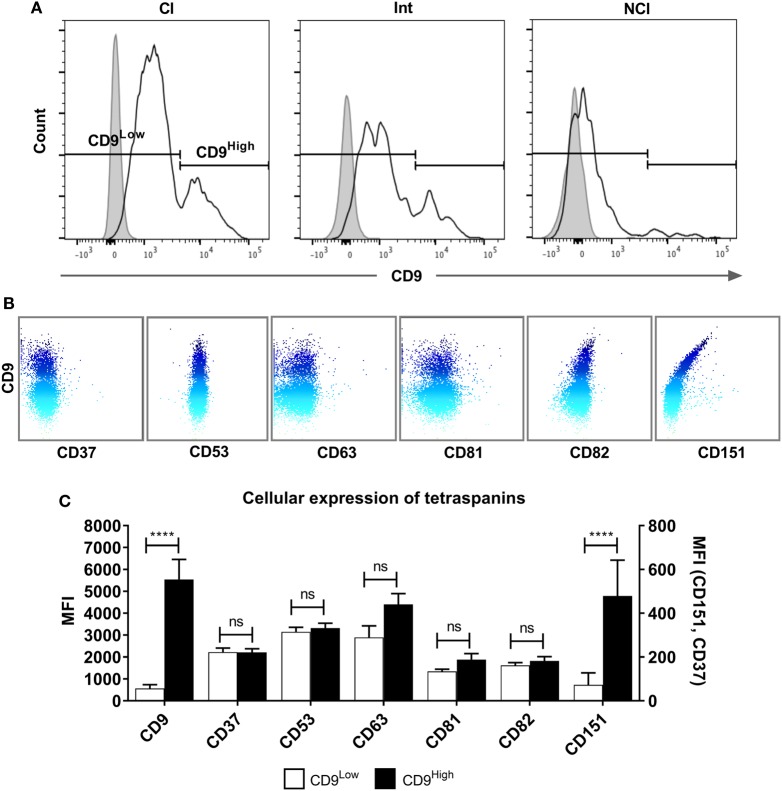
A tetraspanin CD9^High^ subset of classical monocytes. **(A)** Histograms showing the expression of CD9 on freshly isolated classical (Cl), intermediate (Int), and non-classical (NCl) monocyte subsets from a representative donor, with isotype control fluorescence shown as shaded areas. The gating strategy to separate the CD9^High^ and CD9^Low^ populations is indicated by the markers. CD9 was the only tetraspanin in the histograms to show a bimodal peak of expression. **(B)** Dot-plots showing the surface co-expression of tetraspanins on classical subset monocytes with CD9 from a representative donor. Increasing expression of CD9 is indicated by the blue shading. **(C)** Quantification of tetraspanin expression on Cl subset monocytes gated for CD9 high and low expression as shown in panel A. Bars represent the means ± SEM, from 10 different donors. Significance was tested using one-way ANOVA with Sidak’s multiple comparisons test for each pair of columns.

Tetraspanins, particularly CD9, CD81, CD151, and CD63, have been associated with cell fusion in monocytes, developing muscle, and during fertilization (43). We therefore measured the plasma membrane expression of seven common tetraspanins in the freshly purified subsets (Figures 4A,B). The surface expression of tetraspanins on the monocyte subsets shows wide variation, with CD9 and CD37 significantly more highly expressed in the Int subset, in terms of absolute expression levels (MFI) and CD37 is more widely expressed in Int monocytes when expressed as a percentage of the population of cells. After 4 h ConA treatment of adherent monocytes, the expression of all of some of the tetraspanins (CD53, CD82, and in percent positive cells, CD37) declines (Figures 4C,D). In terms of the expression per cell, the MFI values for CD53 and CD82 decrease significantly in all subsets. Interestingly, expression of CD9, often thought to be a negative regulator of fusion and so expected to decrease after ConA treatment, showed no significant changes in any of the subsets (Figures 4C,D). We also identified a population of CD9^High^ cells in the unstimulated Cl subset. Typically, ~75% of Cl monocytes were CD9^Low^ and ~23% CD9^High^ (Figure 5A). Examining the co-expression of other tetraspanins with CD9, the dot-plots show an apparent degree of correlation and statistical analysis confirms that CD151 is significantly elevated in CD9^High^ Cl monocytes (Figures 5B,C). However, when sorted, CD9^High^ Cl monocytes did not show a different ability to fuse compared to CD9^Low^ Cl monocytes (data not shown).

### Effects of Anti-Tetraspanin Antibodies on MGC Formation

**Figure 6:**
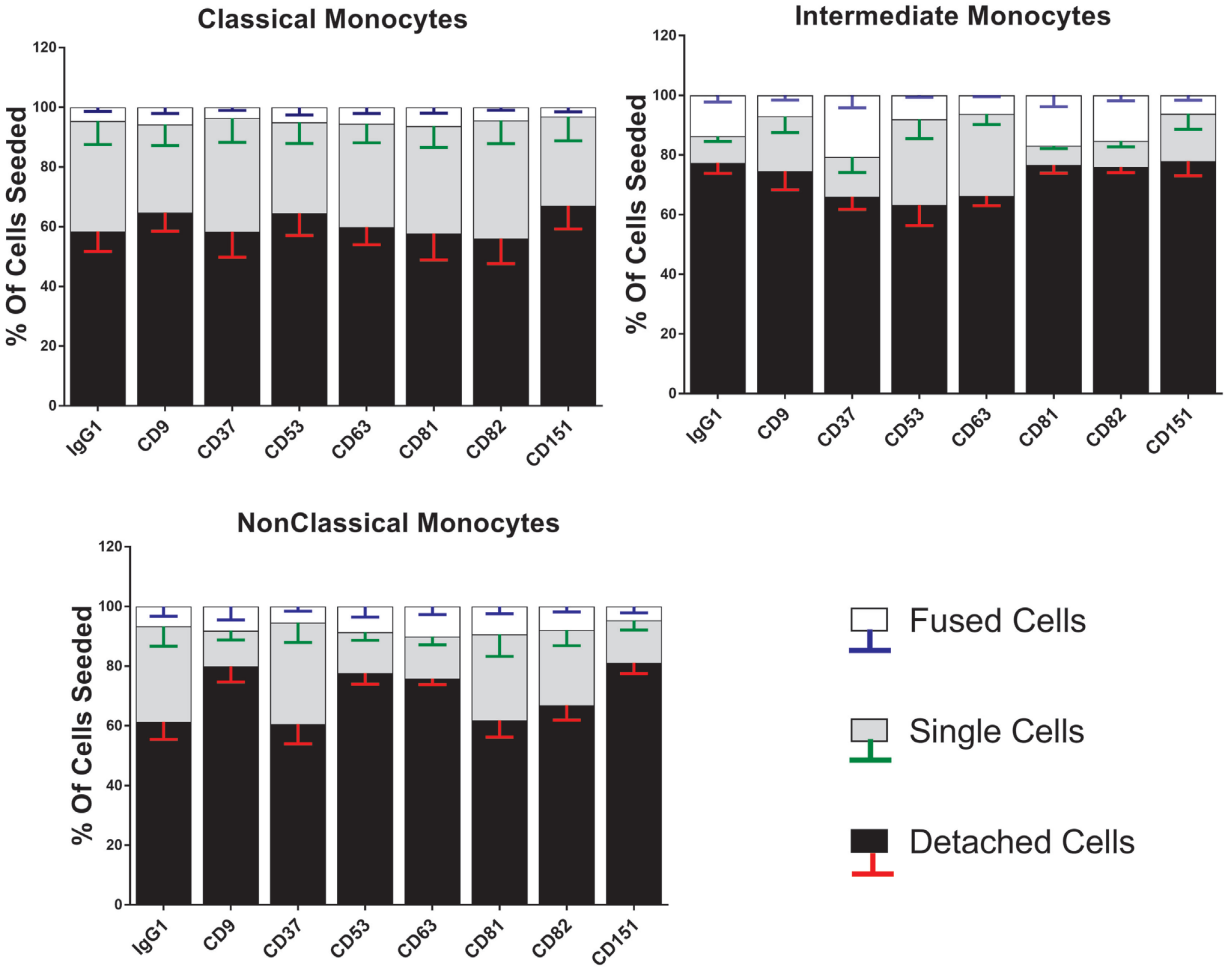
Anti-tetraspanin antibodies do not affect cell fate during concanavalin A (ConA)-induced fusion. The fate of sorted monocyte subsets was determined by counting nuclei at 72 h and expressed as a percentage of the cell numbers originally plated. Bars represent means ± SEM, *n* = 3–8. Significance was tested with a Kruskal–Wallis test with Dunn’s multiple comparisons test comparing the means of the same state within the same time point against the other subsets. Black bars/red error bars: detached cells, gray bars/green error bars: single cells and white bars/blue error bars: fused cells with >3 nuclei.

Anti-tetraspanin antibodies have previously been shown to either positively (anti-CD9, anti-CD81) or negatively (anti-CD63, anti-CD151) affect the size of MGC formed by fusing monocytes (40–42), although the contribution of the different subsets and the types of MGC formed have not been analyzed before. Here, we have used a range of anti-tetraspanin antibodies to investigate their contribution to subset- and MGC type specific effects during monocyte fusion (Table S1B in Supplementary Material). First, we investigated the effects of antibodies on the fate of seeded cells (Figure 6). None of the tetraspanin antibodies caused a significant increase in cell detachment, suggesting that any effects on fusion were not caused by changes in cellular adherence or survival. However, several antibodies (against CD9, CD53, CD63, and CD151) did show a trend toward increased cell detachment.

**Figure 7:**
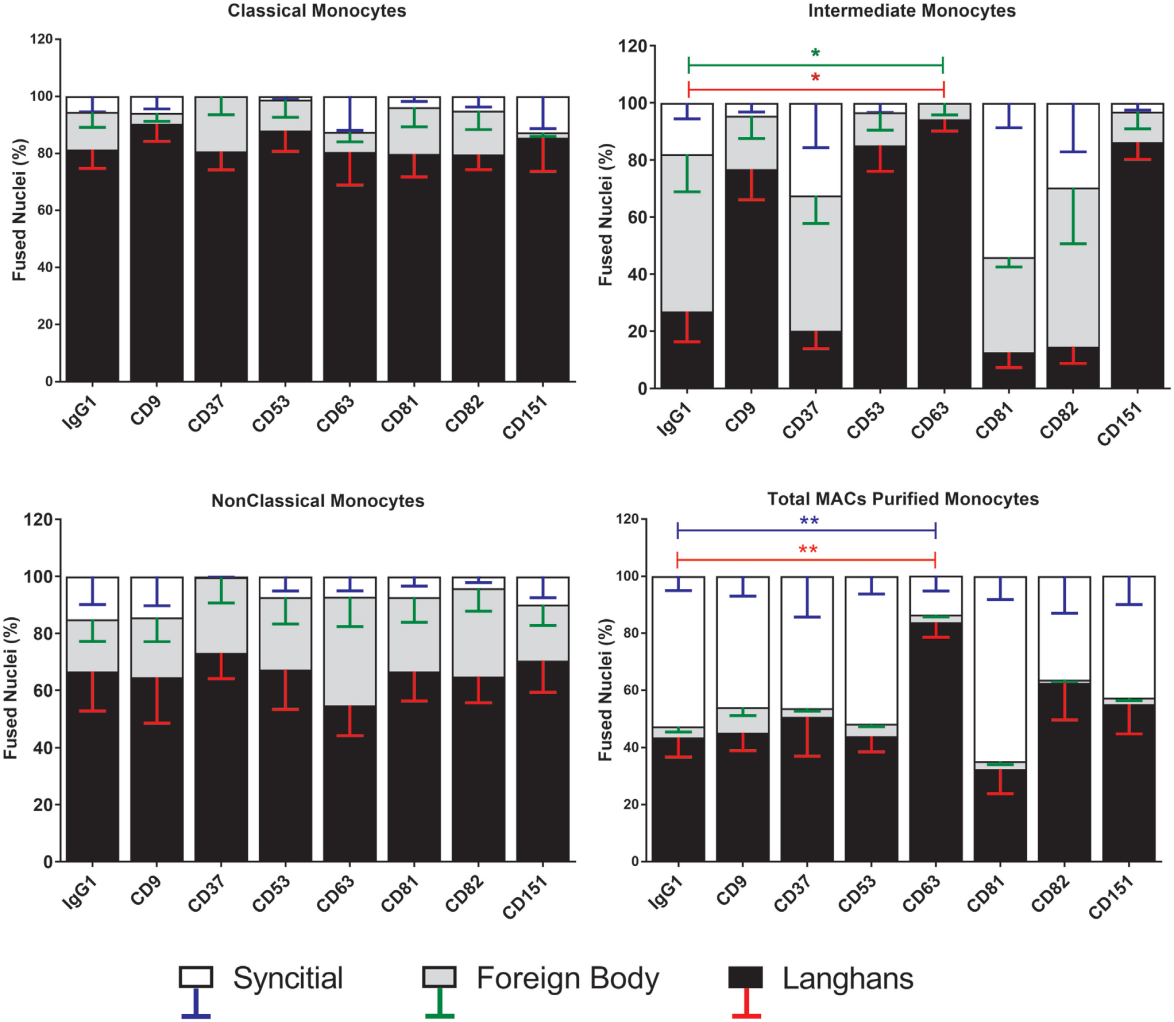
Anti-CD63 antibodies can modulate giant cell morphology only in the intermediate monocyte subset. Purified monocyte subsets were cultured in media containing concanavalin A (ConA) and either an anti-tetraspanin antibody or IgG1 control at 10 µg ml^−1^ for 72 h. Nuclei counted inside each monocyte-derived giant cell (MGC) were ascribed to one of the giant cell types and presented as a percentage of the fused nuclei counted. Bars represent means ± SEM, *n* = 3–8, tested with a Kruskal–Wallis test with Dunn’s multiple comparisons tests comparing the same MGC types between the anti-tetraspanin conditions and the IgG1 control. Black bars/red error bars: Langhans giant cell, gray bars/green error bars: foreign body giant cell and white bars/blue error bars: SGC.

Strikingly, the proportions of each type of MGC produced by fusion of Int monocytes were changed by the anti-tetraspanin antibodies used here (Figure 7) whereas Cl and NCl subsets were not affected. Anti-CD63, in particular caused significant changes in the proportions of MGC formed. It inhibited SGC formation completely and promoted a much higher proportion of LGC to FBGC. Anti-CD9, anti-CD53, and anti-CD151 antibodies exhibited similar effects but the changes did not reach significance. Total (unseparated) MACS-purified monocytes were also significantly affected by only anti-CD63. Anti-CD37 antibody also showed a trend toward the inhibition of SGC formation in Cl and NCl monocytes. Interestingly, the proportions of the various MGC types formed by unfractionated monocytes did not resemble those formed by the isolated subsets, suggesting that interactions between the monocyte subsets can also affect the type of MGC formed (Figure 7). The adherence-purified monocytes responded only to anti-CD9 antibodies, with higher proportions of larger FBGC and SGC (Figure S3 in Supplementary Material).

**Figure 8:**
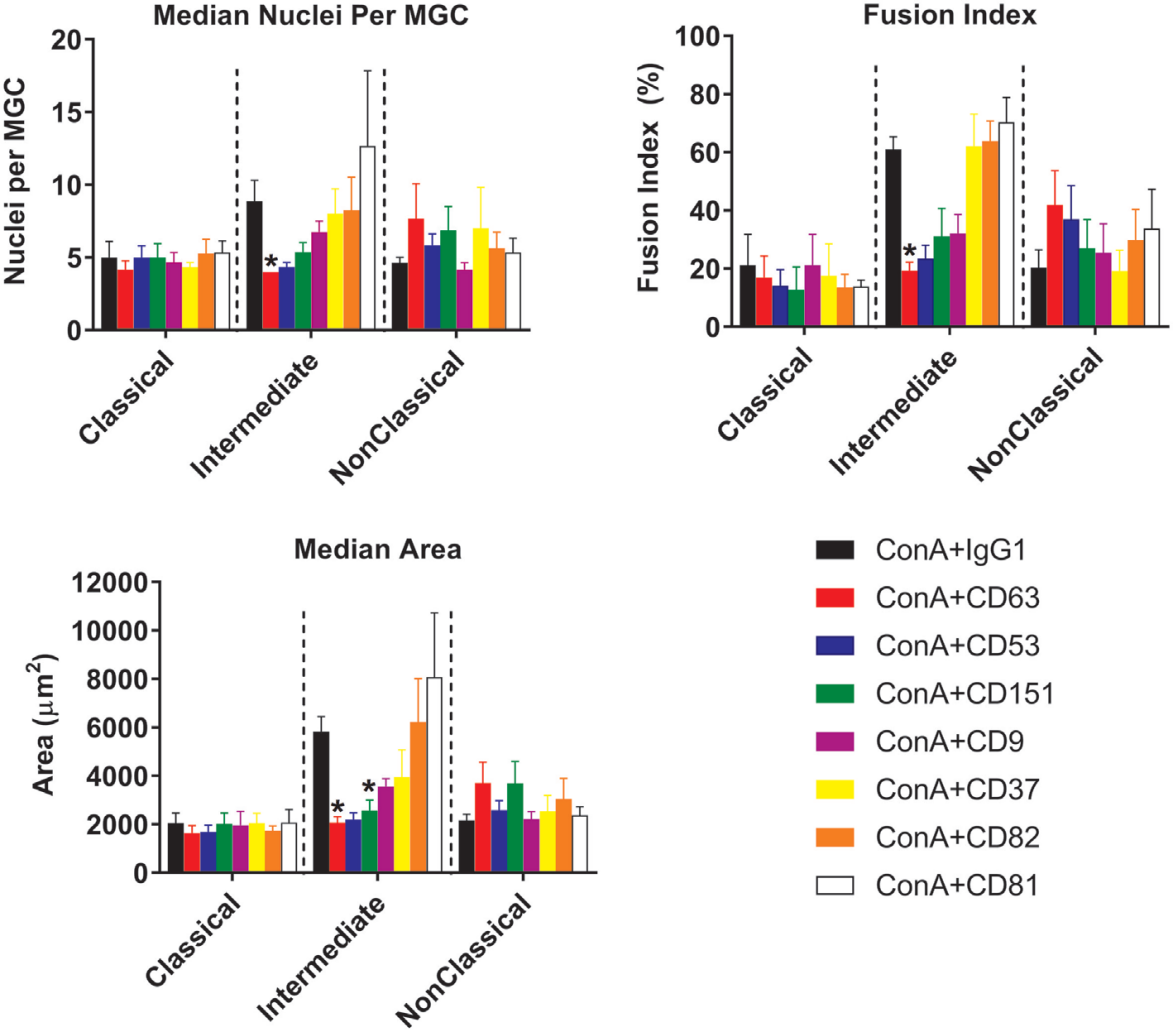
Anti-tetraspanin antibodies inhibit fusion rate and size of giant cells produced by intermediate monocyte subset. Purified monocyte subsets were cultured in media containing (ConA) and either an anti-tetraspanin antibody or IgG1 control at 10 µg ml^−1^ for 72 h. Bars represent means ± SEM, of 3–8 separate experiments. Significance was tested with a Kruskal–Wallis test and a Dunn’s multiple comparisons test comparing the anti-tetraspanin antibody means against the IgG1 control within each subset.

With respect to the sizes of the MGC formed, only Int monocytes were affected by treatment with anti-tetraspanin antibodies. In contrast to previous data on total monocytes purified by adherence (40, 41) and as shown here in Figure S3 in Supplementary Material, anti-CD9 antibodies did not cause an increase in MGC size and have no significant effects on either FI or the number of nuclei per MGC on any of the monocyte subsets. However, anti-CD63 antibodies were found to be consistently inhibitory on all parameters of Int fusion (Figure 8). Anti-CD151 antibody also caused a significant decrease in MGC median area whereas anti-CD53 also inhibited MGC formation but this did not reach significance, *p* = 0.056.

### Expression of Fusion-Related Molecules on Monocyte Subsets

**Figure 9:**
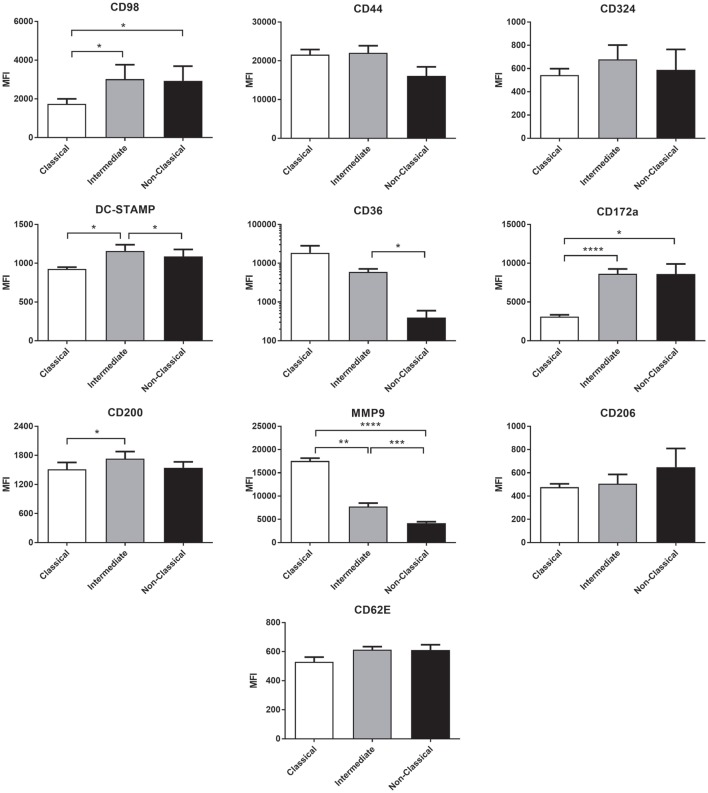
The expression of fusion-related molecules is higher on intermediate monocyte subset. Freshly isolated monocytes were analyzed for fusion protein expression on subsets using flow cytometry. Bars indicate means ± SD from three separate experiments. Significance of difference between subsets was tested with a one-way ANOVA and a Tukey multiple comparisons test.

Many molecules have been associated with monocyte fusion (26–33) and so we examined a panel of 10 membrane proteins for differential expression on the unstimulated subsets. Int monocytes were clearly enriched for a number of these, including DC-STAMP, CD98 CD17a, and CD200 relative to one or both of the other subsets (Figure 9). This overall pattern of fusion molecule expression might explain the greater propensity of the Int subset to undergo ConA-stimulated fusion.

### Cytokine Expression in Fusing Monocytes

**Figure 10:**
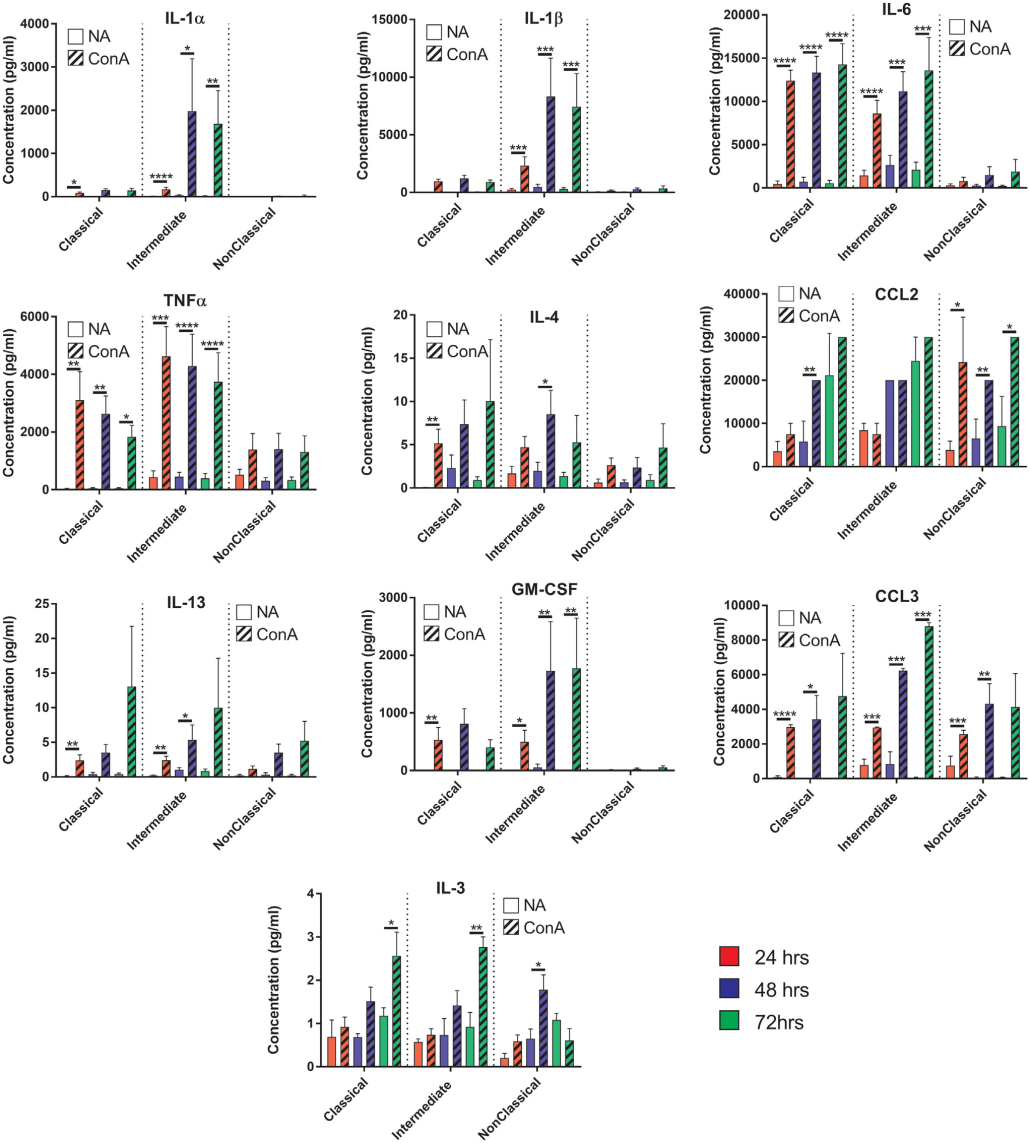
Cytokine production profiles during fusion do not correlate with fusion rate or giant cell morphology. Supernatants from the fusing monocytes were collected and analyzed by ELISA for 15 cytokines relevant to fusion. Clear bars: control (NA), striped bars: concanavalin A (ConA) treated; with each time point [24 (red), 48 (purple), 72 h (green)] presented in adjacent pairs. Bars represent means ± SEM, from eight separate experiments, all tested for significance with a two-way ANOVA with a Sidak’s multiple comparison test comparing the means of control (NA) vs ConA treated monocytes at the same time point within each subset.

To further investigate the mechanism behind the greater fusogenicity of Int monocytes, we analyzed cytokine production during ConA-mediated fusion to determine if fusogenic cytokine production could contribute to this. ConA-stimulated cytokine production was significantly higher in Int monocytes for IL-1α and IL-1β, confirming the higher pro-inflammatory capacity of this subset (Figure 10). However, other cytokines previously identified as being pro-fusogenic, such as CCL2, IL-4 and IL-13, were not elevated in cultures of Int monocytes when compared to the other two subsets. Thus, the Int monocytes appear to generally secrete higher levels of cytokines but no particular cytokine (of those measured) can be described as playing a pivotal role in the control of MGC formation. IFNγ, IL-10, IL-17A, CCL5, and VEGF were also tested but were either not detected or were not significantly different from unstimulated controls.

## Discussion

Here, we demonstrate for the first time that human monocyte subsets show very different propensities to form MGC in response to ConA stimulation. The Int subset fused faster and formed more of the larger FBGC and SGC types while the Cl fused to form mostly the smaller LGC.

### Monocyte Purification Methods

Previous studies on the role of tetraspanins in ConA-induced monocyte fusion used cells purified by adherence (40, 41), which have a very different pattern of fusion and anti-tetraspanin antibody sensitivity to the MACS-purified total monocytes used in this report (Figures 3 and 7; Figure S3 in Supplementary Material). The MACS technique specifically enriches monocytes whereas the adherence method relies on the ability of cells to rapidly adhere to plastic surfaces and some contaminating T and NK cells may be present. In addition, Int and NCl subsets are less adherent than Cl within the first 24 h after isolation, and so in the previous studies more of the initially adherent low-fusing Cl and fewer of the high-fusing Int and NC subsets may have been present. Although Int were observed to be the most fusogenic of the subsets (Figure 3A), total MACS-purified monocytes stimulated with ConA showed even higher fusion parameters with many more FBGC and SGC (Figure 7). This suggests that fusion potency is increased by interaction between the subsets.

### Monocytes Subsets and Fusion

The histological type of MGC formed by each subset has been quantified, with the Int and NCl showing a greater capacity to form the larger FBGC and SGC types. This has implications for the treatment of medical implant rejection as it is clearly the Int and NCl subsets that form the larger MGC associated with foreign body rejection. Interestingly, the Int subset is increased in the blood of sarcoidosis patients (15), a condition characterized by granulomas in which FBGC and LGC are present (48). The increased ability of the Cl subset to form LGC could indicate that they are specialized in responding to mycobacterial infections, as LGC are commonly found in granulomatous infections *in vivo* (19).

### Tetraspanin Expression on Subsets

Our data for the expression of CD9, CD53, CD63, and CD81 does not correlate directly with that of Tippett and co-workers (44), who observed higher percentages of cells expressing CD9 and CD63 overall in each subset. Furthermore, they ranked the intensity of surface expression of CD9 on the subsets as Cl > Int > NCl, CD53 as NCl > Int > Cl, and CD81 as NCl > Int > Cl, whereas here all three tetraspanins were found to be highest on the Int subset. However, the CD14/CD16 gating strategy used by Tipett and co-workers may not have been as stringent as here and so the distinction between the subsets may be less clear. In addition, they did not mention any techniques to remove CD16+ NK cells, which overlap with NCl in CD14^+^/CD16^+^ populations. Overall, we found the Int subset expressed the highest levels of all tetraspanins in freshly purified monocytes except for CD82, which was significantly higher in the Cl subset. The addition of ConA induced significant decreases in the level of CD53 and CD82 and a decrease in the percentage of cells expressing CD37, CD53, and CD82. It appears that ConA induces rapid downregulation of these tetraspanins from the cell surface, although there is no obvious correlation with fusion. Other tetraspanins implicated in fusion, such as Tspan13 and Tspan5, show decreased or increased expression, respectively, in response to RANKL stimulation (39). Tarrant and co-workers (49) showed that Tspan32 knockout mice produced T-cells that became hyper-stimulated by ConA. In future studies, it would be interesting to investigate the effects of ConA on further members of the tetraspanin family. The Cl monocyte subset showed an intriguing bimodal expression of CD9, with nearly 25% of this subset (and thus ~20% of total monocytes) having a significantly higher surface expression of this tetraspanin. Co-expression analysis of the tetraspanins showed a positive correlation between CD9 and CD151 expression on Cl. While CD9^High^ Cl showed no increase in fusion potential, it would be interesting in future work to investigate other functions of these cells, such as their propensity for extravasation.

### Anti-Tetraspanin Antibodies and Fusion

The Int subset showed clear significant decreases in fusion parameters and MGC types produced when cultured in the presence of anti-CD63. Anti-CD63 also significantly inhibited MGC formation by total MACS-purified monocytes in response to ConA, in agreement with previous data (41). No inhibition by anti-CD63 was observed for the Cl and NCl subsets, however, suggesting that fusion may be orchestrated differently in the subsets. It is also possible that the lower baseline fusion rates of Cl and the NCl subsets could be masking any notable reductions by these antibodies. It is not clear from the present work if anti-CD63 treatment is directly affecting cell fusion. CD63 knockdown causes arrested motility due to decreased actin polymerization by engaged E-cadherin (50). Therefore, it is possible that the decrease in fusion is a result of arrested mobility and not interference with the fusion mechanism. The lack of a change in the expression level of CD63 during ConA stimulation suggests that antibody might be modulating function by sequestering CD63 away from partner proteins, for example, or by clustering molecules together to activate signaling. Further work is required to distinguish between these possibilities.

Interestingly, antibodies against CD9, CD53, CD63, and CD151 did show a trend toward increased cell detachment in the NCl subset and this pattern was also seen in the effects on Int subset MGC types. This suggests that these tetraspanins might have a role in monocyte behavior but that antibodies are not ideal tools to study this role.

### Fusion-Related Membrane Proteins

We hypothesized that increased fusion and sensitivity to anti-CD63 antibody in the Int subset might be due to changes in the expression of membrane proteins known to play a role in fusion, many of which are also known to be partners of tetraspanins. The high-fusing Int monocytes showed generally high levels of the fusion-mediating molecules DC-STAMP, CD172a, CD200, and CD62E and low levels of MMP9 and CD36 relative to the other subsets. DC-STAMP, the only molecule significantly higher in Int than in both of the other subsets, has been shown to be essential for cell–cell fusion in osteoclasts and FBGCs (32, 33). CD200, significantly higher in Int than Cl, is expressed in monocytes after the induction of fusion (26). SIRPα/CD172a/MFR, also higher in Int than Cl, has been shown to be essential for MGC formation (27). MMP9 has been shown to be involved in mouse MGC formation *in vivo* and in response to IL-4 *in vitro* (31) but was found here to be significantly lower in Int monocytes than in Cl. CD36, a phosphatidylserine and lipid binding protein, has been shown to have a role in cytokine-induced MGC but not in osteoclast generation (29). Cl and Int monocytes had higher levels of CD36 than NCl, but as Cl and NCl have similarly low fusogenic potential, this suggests that CD36 expression is not specifically related to higher fusion rates in the Int subset. CD62E (E-selectin) has been implicated in osteoclast formation (51) and MGC formation driven by *B. pseudomallei* infection of U937 cells (30). While it is not significantly more highly expressed in Int monocytes than Cl, CD62E is expressed at a similar level on the lower fusing NCl monocytes. Taken together, however, our data indicate that differences in the expression of multiple fusion-related molecules might be related to the greater fusion capacity of Int monocytes.

### Cytokine Production During Fusion

We hypothesized that different levels of cytokine production during ConA stimulation might play a role in the differences in fusion between subsets but the lack of a clear pattern suggests that this is not the primary driver of the variation between monocyte subsets. However, the subsets do show remarkably different cytokine profiles during fusion. The Int and Cl secreted pro-apoptotic cytokines (TNFα) within the first 24 h followed by an increase in pro-inflammatory cytokines (IL-1α, IL-1β, and IL-6) by 48 h. IL-1α and IL-1β have been shown to be released from cells undergoing apoptosis (52) and we also observed at these same time points that a large number of monocytes (57–65%) were dead or detached. This could suggest that many ConA-stimulated monocytes undergo apoptosis and the release of internal IL-1 is a necessary step to generate the fusogenic cytokines. However, the NCl subset achieved greater fusion rates than the Cl monocytes but did not release high levels of IL-1α, IL-1β, or TNFα, suggesting that apoptosis of some cell types is not a pre-requisite for fusion.

In summary, we have shown that the various monocyte subsets differ in their capacity to form MGC in response to ConA, with the Int subset showing greatest propensity for fusion. For this subset, there is evidence that the tetraspanin CD63 may be involved in the process. It is interesting to speculate that the increased fusogenic potential of Int moncytes may relate to their roles in granuloma formation in infectious and inflammatory conditions *in vivo*.

## Author Contributions

PM, LP, and SW planned experiments and wrote the manuscript; BM and S-MO performed experiments; TC designed and performed experiment and analyzed data.

## Conflict of Interest Statement

The authors declare that the research was conducted in the absence of any commercial or financial relationships that could be construed as a potential conflict of interest.

## Supplementary Material

Figure S1 FACS gating strategy of monocyte subsets from magnet-activated cell sorting-enriched fraction. Pseudocolour/contour plots showing the FACS gating strategy for sorting monocyte subsets. First, monocytes were broadly selected by their forward and side scatter profiles followed by singlet gating. NK cells were removed by selecting for CD56^−^ cells. Second, CD14^++^CD16^−^ (Classical), CD14^++^CD16^+^ (Intermediate), and CD14^+^CD16^+^ (non-classical) monocytes were gated as shown and sorted. The purity of the sorted populations was always >90%.

Figure S2 Morphologies of the three monocyte-derived giant cell (MGC) types observed during fusion assays. A representative low magnification image with nuclei in red and F-actin in blue. Langhans giant cells can be identified by their horseshoe or ring-shaped nuclear arrangement and are typically the smallest. Foreign body giant cells (FBGC) are larger and contain more nuclei in a stacked central cluster. Syncytial giant cells (SGC) are the largest, have heterogeneous spreading of the membrane and unevenly distributed nuclei within.

Figure S3 Monocyte-derived giant cell (MGC) types generated from adherence-purified total monocytes. The MGC types generated from total monocytes purified by adhesion cultured for 72 h in concanavalin A (ConA) media and corresponding anti-tetraspanin antibody. Fused nuclei were tallied into either Langhans giant cell, FBGC, or SGC depending on what MGC type they were found in and expressed as a percentage of all fused nuclei. Bars represent the mean ± SEM, with data from four separate experiments. Tested with a Dunn’s multiple comparison test; comparing the mean ranks of each MGC type to the IgG1 + ConA control (**p* < 0.05).

## Abbreviations

ConA, concanavalin A lectin; MGC, multinucleated giant cell; Cl, classical monocyte sybset; NCl, non-classical monocyte subset; Int, intermediate monocyte subset; LGC, Langhans giant cell; SGC, syncytial giant cell; FBGC, foreign body giant cell; FI, fusion index; MACS, magnet-activated cell sorting; DC-STAMP, dendrocyte-expressed seven transmembrane protein; SEM, scanning electron microscopy; MFI, median fluorescence intensity; MMP9, matrix metallopeptidase 9.
