# Supplementary figures and images for: Monocyte Subsets Have Distinct Patterns of Tetraspanin Expression and Different Capacities to Form Multinucleate Giant Cells
Source: Front Immunol. 2018 Jun 8;9:1247. doi: 10.3389/fimmu.2018.01247 (PMC6002745; doi:10.3389/fimmu.2018.01247)

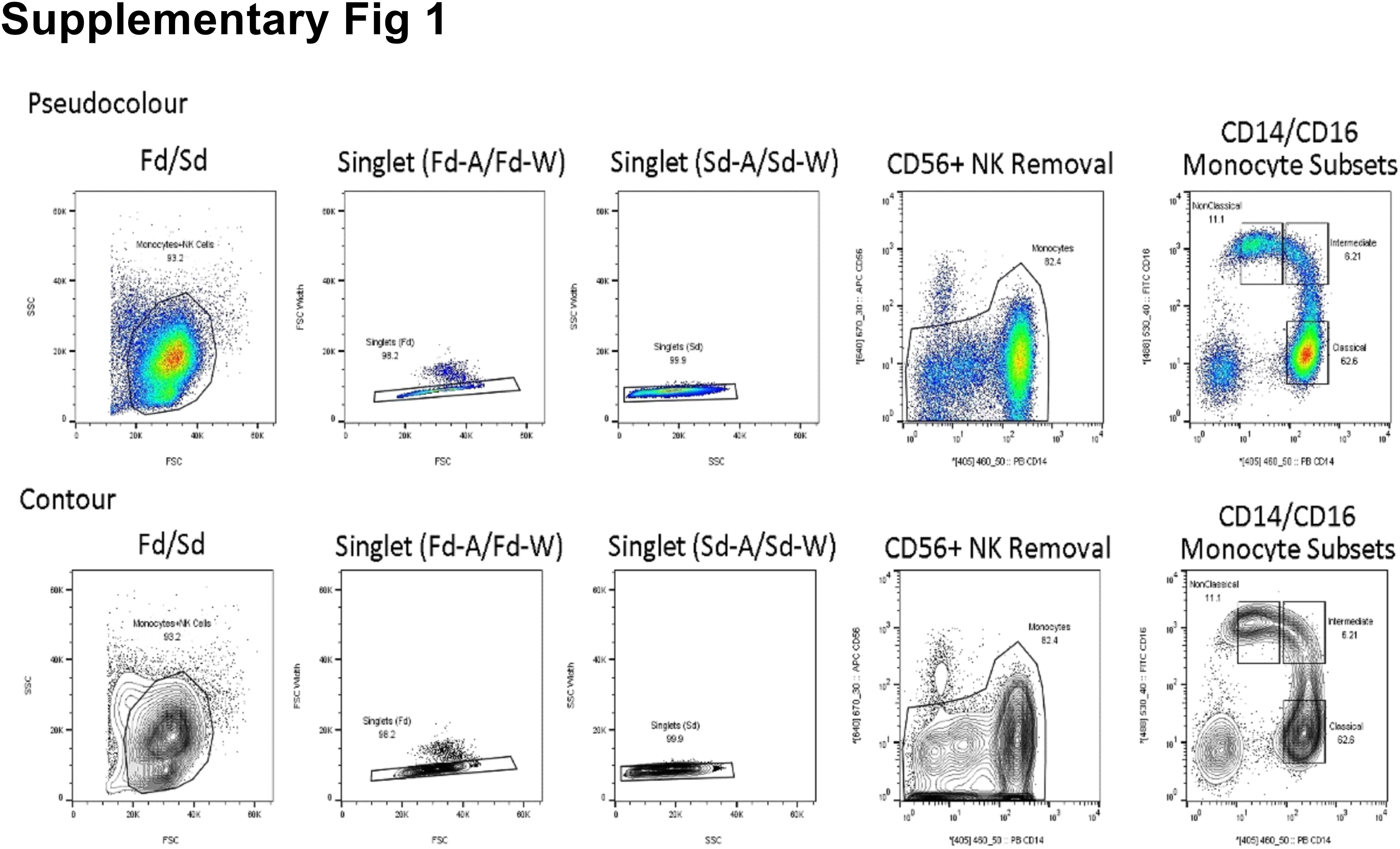

Supplement: Figure S1 — FACS gating strategy of monocyte subsets from magnet-activated cell sorting-enriched fraction. Pseudocolour/contour plots showing the FACS gating strategy for sorting monocyte subsets. First, monocytes were broadly selected by their forward and side scatter profiles followed by singlet gating. NK cells were removed by selecting for CD56− cells. Second, CD14++CD16− (Classical), CD14++CD16+ (Intermediate), and CD14+CD16+ (non-classical) monocytes were gated as shown and sorted. The purity of the sorted populations was always >90%. [file Image_1.tif]

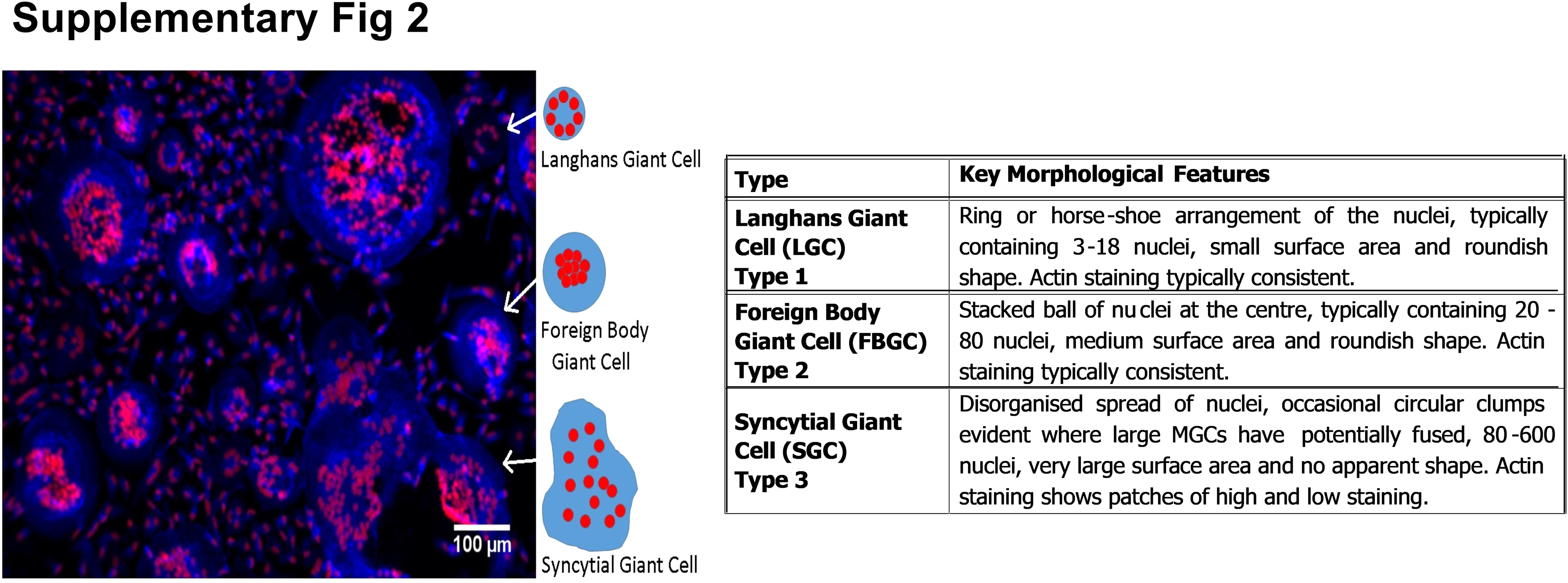

Supplement: Figure S2 — Morphologies of the three monocyte-derived giant cell (MGC) types observed during fusion assays. A representative low magnification image with nuclei in red and F-actin in blue. Langhans giant cells can be identified by their horseshoe or ring-shaped nuclear arrangement and are typically the smallest. Foreign body giant cells (FBGC) are larger and contain more nuclei in a stacked central cluster. Syncytial giant cells (SGC) are the largest, have heterogeneous spreading of the membrane and unevenly distributed nuclei within. [file Image_2.tif]

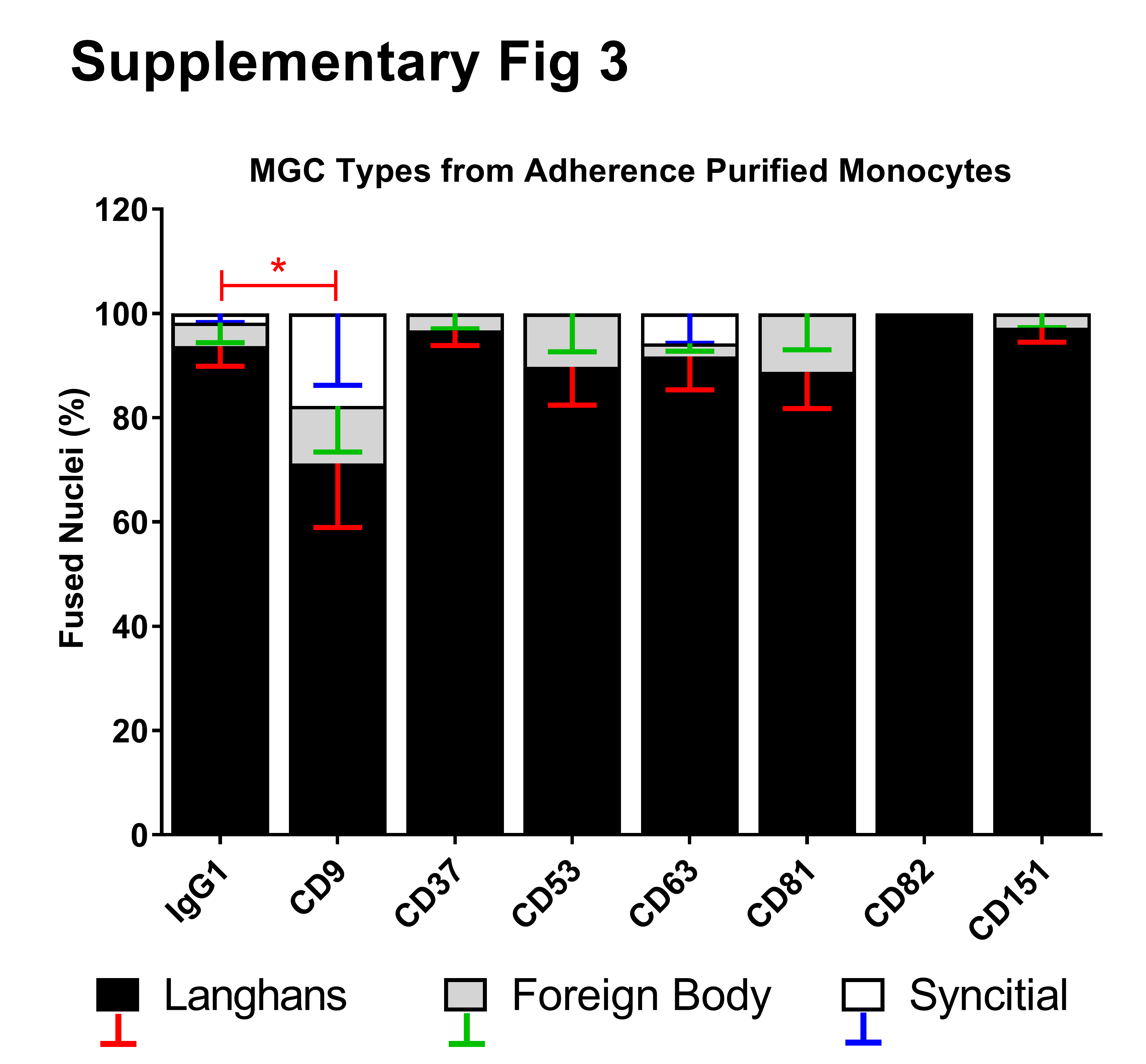

Supplement: Figure S3 — Monocyte-derived giant cell (MGC) types generated from adherence-purified total monocytes. The MGC types generated from total monocytes purified by adhesion cultured for 72 h in concanavalin A (ConA) media and corresponding anti-tetraspanin antibody. Fused nuclei were tallied into either Langhans giant cell, FBGC, or SGC depending on what MGC type they were found in and expressed as a percentage of all fused nuclei. Bars represent the mean ± SEM, with data from four separate experiments. Tested with a Dunn’s multiple comparison test; comparing the mean ranks of each MGC type to the IgG1 + ConA control (*p < 0.05). [file Image_3.tif]
