# Supplementary material for: Monocyte Subsets Have Distinct Patterns of Tetraspanin Expression and Different Capacities to Form Multinucleate Giant Cells
Source: Front Immunol. 2018 Jun 8;9:1247. doi: 10.3389/fimmu.2018.01247 (PMC6002745; doi:10.3389/fimmu.2018.01247)
Supplement: Supplementary file 4 [file Table_1.docx]

**Supplementary Table 1A Flow cytometry antibodies**

| **Antigen** | **Supplier** | **Clone** |
| --- | --- | --- |
| CD98 | ImmunoTools | MEM108 |
| CD44 | eBioScience | IM7 |
| CD324 | eBioscience | DECMA-1 |
| DC-STAMP | Novus Biologicals | 788524 |
| CD36 | Biolegend | 5-271 |
| CD172a | AbDSerotec | 15-414 |
| CD200 | Miltenyi Biotec | OX104 |
| CD62E FITC | eBioscience | CL2 |
| CD206 | Miltenyi Biotec | DCN228 |
| MMP9 | R&D Systems | 56129 |
| CD14-PECF594 | BD Biosciences 5 | MfP9 |
| CD14-eFluor450 | eBioscience | 61D3 |
| CD56-APC | BD Biosciences | NCAM16.2 |
| CD16-PECy7 | BD Biosciences | 3G8 |
| CD16-PE-Vio770 | Miltenyi Biotec | VEP13 |
| CD9-biotin | Abcam | MEM-61 |
| Streptavidin APC-Cy7 | Biolegend | - |
| CD37-APC | eBioscience | MB-1 |
| CD53-CF405M | Abcam | HI29 |
| CD63-PerCP | Abcam | MEM259 |
| CD81-AF700 | Novus | 1D6 |
| CD82-PE | Abcam | B-L2 |
| CD151-FITC | Abcam | 11G5a |

**Supplementary Table 1B Fusion assay antibodies**

| **Antibody** | **Source** | **Clone** |
| --- | --- | --- |
| CD9 | Gift of Peter Andrews, University of Sheffield, UK | 602.29 |
| CD37 | Gift of Martin Glennie, University of Southampton, UK | WR17 |
| CD53 | Abcam | MEM53 |
| CD63 | Developmental Studies Hybridoma Bank | H5C6 |
| CD81 | Abcam | 1D6 |
| CD82 | Abcam | B-L2 |
| CD151 | Gift of Leonie Ashman, University of Newcastle, NSW | 14A2.H1 |

Table 1. Antibodies used for analysis of the monocyte subsets and membrane protein expression
